# Stage-Dependent Expression and Up-Regulation of Trypanothione Synthetase in Amphotericin B Resistant *Leishmania donovani*

**DOI:** 10.1371/journal.pone.0097600

**Published:** 2014-06-05

**Authors:** Asif Equbal, Shashi Shekhar Suman, Shadab Anwar, Krishn Pratap Singh, Amir Zaidi, Abul Hasan Sardar, Pradeep Das, Vahab Ali

**Affiliations:** 1 Laboratory of Molecular Biochemistry and Cell Biology, Department of Biochemistry, Rajendra Memorial Research Institute of Medical Sciences, AgamKuan, Patna, India; 2 Department of Molecular Biology, Rajendra Memorial Research Institute of Medical Sciences, AgamKuan, Patna, India; Federal Institute for Vaccines and Biomedicines, Germany

## Abstract

Kinetoplastids differ from other organisms in their ability to conjugate glutathione and spermidine to form trypanothione which is involved in maintaining redox homeostasis and removal of toxic metabolites. It is also involved in drug resistance, antioxidant mechanism, and defense against cellular oxidants. Trypanothione synthetase (TryS) of thiol metabolic pathway is the sole enzyme responsible for the biosynthesis of trypanothione in *Leishmania donovani*. In this study, TryS gene of *L. donovani* (LdTryS) was cloned, expressed, and fusion protein purified with affinity column chromatography. The purified protein showed optimum enzymatic activity at pH 8.0–8.5. The TryS amino acids sequences alignment showed that all amino acids involved in catalytic and ligands binding of *L. major* are conserved in *L. donovani*. Subcellular localization using digitonin fractionation and immunoblot analysis showed that LdTryS is localized in the cytoplasm. Furthermore, RT-PCR coupled with immunoblot analysis showed that LdTryS is overexpressed in Amp B resistant and stationary phase promastigotes (∼2.0-folds) than in sensitive strain and logarithmic phase, respectively, which suggests its involvement in Amp B resistance. Also, H_2_O_2_ treatment upto 150 µM for 8 hrs leads to 2-fold increased expression of LdTryS probably to cope up with oxidative stress generated by H_2_O_2_. Therefore, this study demonstrates stage- and Amp B sensitivity-dependent expression of LdTryS in *L. donovani* and involvement of TryS during oxidative stress to help the parasites survival.

## Introduction

Leishmaniasis is a spectrum of disease affecting more than 12 million people worldwide caused by protozoan parasites of the genus *Leishmania.* Leishmaniasis is divided into three major types based on the body parts or organs affected, known as cutaneous, mucocutaneous and visceral. The visceral leishmaniasis (VL, Kala-azar) is a symptomatic infection of liver, spleen, and bone marrow and is fatal, if left untreated. The global estimates for the incidence and prevalence of kala-azar cases per year are 0.5 and 2.5 million, respectively (WHO report, 1998) and it poses a major health problem in Bihar, which accounts for nearly 90% of the total cases in India [1]. The available treatment for VL is only chemotherapy and mainly depends on sodium stibogluconate (SAG, first line drug) but nearly 65% cases showed resistance against it and hence, no more preferred to use for the treatment of VL patients. Second line drug of choice Amphotericin B (Amp B) also showed relapse and developed resistance [2], [3]. However, available drugs for leishmaniasis are far from satisfactory because they are highly toxic, cost ineffective, poor efficacy, or loss of effectiveness due to development of drug resistance after prolonged use [4], [5]. So, for more satisfactory treatment of leishmaniasis, targeting of metabolic pathways that are crucial for parasite viability or infectivity, and absent or differ significantly from those found in the mammalian host, may provide clues for rational drug design [6]. Such a unique metabolic pathway present in *Leishmania* along with other trypanosomatids, is biosynthesis of trypanothione, T(SH)_2_ [7] which replaces glutathione (GSH) functions in trypanosomatids [8] and maintained in the reduced state by the flavoenzyme trypanothione reductase (TryR) at the expense of NADPH [9]. It is a peptide amine conjugate synthesized in two consecutive steps using two molecules each of ATPs, GSH, and one molecule of spermidine (Spd) catalyzed by trypanothione synthetase (TryS; EC 6.3.1.9). Spd is synthesized by polyamine pathway [10] and is involved in cellular proliferation and differentiation, whereas GSH is a tripeptide synthesized by γ-glutamylcysteine synthetase (γ-GCS) and involved in defense against oxidative stress [11]–[13]. The essentiality of TryS has been established by dsRNA interference knock-down in *T. brucei* parasites which declined T(SH)_2_ and glutathionylspermidine (Gsp) level, while the level of GSH increased with concomitant growth arrest, impaired antioxidant capacity and infectivity, and ultimately cell death [14]. This observation demonstrated that depletion of TryS is sufficient to impair the defence against oxidative challenge because this gene is present upstream in thiol metabolic cascade [15] and GSH is unable to replace T(SH)_2_ functions. The biosynthesis of T(SH)_2_, however appears to differ between trypanosomatids genera, e.g., in the insect pathogen *Crithidia fasciculata*, two distinct enzymes were reported to catalyze the stepwise ligation of two GSH molecules to Spd [16]–[18], whereas in human pathogen *T. cruzi* [5], *T. brucei* [5], [19], and *L. major* [20], a single enzyme (TryS) could catalyze both steps of T(SH)_2_ biosynthesis. However, recent genome analysis showed that *T. cruzi, L. infantum, L. mexicana* retained GSPS full length gene, but so far, not yet characterized. Similarly, *L. major* and *L. braziliensis* possess pseudogene of GSPS [21] but *L. donovani, L. amazonensis* and *T. brucei* lack GSPS and T(SH)_2_ biosynthesis solely depends on TryS (http://tritrypdp.org/tritrypdp). So, TryS is expected to be essential in trypanosomatids, where an active GSPS is absent. Recently, TryS was characterized in non-pathogenic strain (UR6) of *L. donovani* and some inhibitors of this enzyme showed leishmanicidal activity suggesting an attractive and potential drug target [22]. TryS is present as a single copy gene and the encoded protein is a bifunctional enzyme having synthetase activity in the central domain and amidase activity at their N-terminal and C-terminal domain (20–25 amino acids). The synthetase activity is responsible for T(SH)_2_ biosynthesis by catalytic mechanism, whereas, amidase activity converts the T(SH)_2_ back to GSH and Spd [20], [23], [24].

T(SH)_2_ plays a pivotal role in a number of processes such as intracellular thiol redox balance [25], deoxyribonucleotide synthesis [26], and resistance to trivalent antimonials [27]. *In vitro* selected, SAG resistant parasites have higher levels of T(SH)_2_ than susceptible which alleviates the reactive oxygen species (ROS) generated by the macrophages during infection or drug pressure. Interestingly, trypanosomatids lack catalase and selenium dependent glutathione peroxidases which rapidly metabolizes H_2_O_2_ in other organisms [28], [29]. Their unique antioxidant mechanism against peroxide metabolism relies on the T(SH)_2_; demonstrated by the generation of γ-GCS knockout promastigotes that produced lower T(SH)_2_ level and increased *in vitro* susceptibility to oxidative stress, ultimately leading to decreased survival of parasites inside the activated macrophage [30], [31]. Intracellular killing of *Leishmania* mainly depends on ROS, RNS, & peroxynitrile [21], [32], [33] whereas parasites have evolved mechanisms to overcome these toxic free radicals damage. *Leishmania* parasites use intracellular thiols, T(SH)_2_, ovothiol, Fe^2+^-SOD, Hsp70, and peroxiredoxin [34]–[36] to overcome ROS and NOS during their life cycle. It involves the participation of thiol presenting molecules, such as T(SH)_2_, tryparedoxin (TXN), & tryparedoxin peroxidase (TXNPx) [21], [33] and polyamines [37], [38]. Parasites usually show higher expression of ornithine decarboxylase (ODC) during oxidative stress and hence, increased polyamine metabolism. The opposing synthetase and amidase activity of TryS was proposed to regulate polyamine level in response to polyamine availability and growth phase [5], [18], [19]. Therefore, it is important to know whether the TryS has any role in evading the effects of ROS generation in *L. donovani* promastigotes as it is essential for their survival.

In this manuscript, we have investigated the differential regulation of TryS at both transcriptional and translational level in exponential vs. stationary phase as well as drug sensitive vs. resistant promastigotes and found that LdTryS is overexpressed in stationary phase and Amp B resistant parasites. Moreover, H_2_O_2_ treatment showed an increase in LdTryS expression in a dose dependent manner suggesting its role in coping oxidative stress. This is the first report of stage and stress-dependent expression of TryS in sensitive and Amp B resistant clinical isolates of *L. donovani* promastigotes.

## Materials and Methods

### Ethical Statement

For animals procedures used were reviewed and approved by the Animal Ethical Committee, Rajendra Memorial Research Institute of Medical Sciences, Indian Council of Medical Research. The RMRIMS, ICMR follows “The Guide for the Care and Use of Laboratory Animals”, 8th edition by the Institute for Laboratory Animal Research.

All human blood samples used in this study were collected after obtaining written informed consent from the study participant under the protocol activity approved by the Institutional Ethics Committee (IEC) of RMRIMS. The written consent was approved by the same committee and recorded in the Department of Clinical Medicine, RMRIMS. The IEC of RMRIMS follows the guidelines of Medical Ethics Committee of Indian Council of Medical Research (ICMR), Department of Health Research, Ministry of Health and Family Welfare, Govt. of India.

### Chemicals and Reagents

All chemicals of analytical grade were purchased and used from Sigma-Aldrich, Amresco (USA), and USB (USA) unless otherwise stated. Ni^2+^-NTA agarose matrix and Gel extraction kit were purchased from Qiagen. Plasmids and restriction enzymes were purchased from Novagen and Fermentas. Culture mediums M199, Schneider’s Insect Medium and RPMI-1640 used were from Hyclone and Sigma. Spermidine was purchased from Duchefa biochemie, Netherlands.

### Clinical Isolates and Parasites Culture

Clinical isolates of Amp B resistant strains were obtained from the splenic aspirates of VL patients in the indoor ward facility of Rajendra Memorial Research Institute of Medical Sciences, Patna, Bihar, India. The collected splenic aspirates were incubated in biphasic (N, N, N, medium) supplemented with HBSS (Invitrogen). The amastigotes from splenic aspirates were transformed into promastigotes and the isolates initially were incubated in the Schneider’s Insect gut medium (pH-7.4) supplemented with10% heat inactivated fetal bovine serum (HIFBS). These isolates were finally maintained in M199 medium supplemented with 10% HIFBS. Standard sensitive *L. donovani* strains Ag83 (MHOM/IN/83/Ag) and Dd8 (MHOM/IN/80/Dd8) were cultured in the medium M199 supplemented with 10% HIFBS and 25 mM HEPES buffer (pH 7.2), 100 units/ml penicillin and 100 µg/ml streptomycin. Culture was initiated at 1×10^5^ parasites/ml and grown at 24±1°C in BOD incubator for 4–5 days before sub culturing (late log phase).

### *In vitro* Drug Sensitivity Assay

To determine in vitro drug sensitivity of clinical isolates as well as standard *L. donovani* strains, triplicate culture containing different concentration of drugs (Amp B or SAG) were seeded at 1×10^6^ parasites/ml in M199 medium (supplemented with 10% HIFBS). Cell densities were determined microscopically after culturing for 24 hrs, viable cells counted on a haemocytometer by the trypan blue exclusion method and 50% effective concentration (EC_50_) values were determined for both drugs sensitive and resistant strains. Interestingly, Amp B resistant isolates were also unresponsive to SAG and show higher EC_50_ values for both Amp B and SAG as determined by *in vitro* drug sensitivity assay [39]. The cytotoxicity was also analyzed by cell proliferation reagent WST-1 (Roche) assays; briefly, 100 µl cell suspension (1×10^6^ parasites/ml) were cultured in 96-well plate in the presence of various concentrations of Amp B for 24 hrs, 10 µl WST-1 added in each well, incubated for 1 hr and ELISA plates read at 450, 655 nm in dual mode. The experiments were repeated three times in duplicate.

### Isolation of DNA and RNA

Total RNA was extracted from 1×10^8^ *L. donovani* promastigotes using Trizol method (Invitrogen), according to the manufacturer’s instructions. Total DNA was isolated from promastigotes using phenol/chloroform/isoamyl-alcohol method (25∶24∶1, v/v) followed by ethanol precipitation, as described [40]. The quality and quantity of DNA and RNA were assessed using agarose, formamide gel electrophoresis and spectrophotometer, respectively.

### PCR Amplification and Cloning of LdTryS

Based on the nucleotide sequence of the protein-encoding region of the putative *Leishmania* trypanothione synthetase genes (*LdTryS,* accession number CAD 23679); primers (shown below) were designed to clone LdTryS in vector pET-15b with a histidine tag at the amino terminus. The *LdTryS* ORF was amplified from genomic DNA with a sense (5′-CCTCAT*ATG*TCGTCTCTGCCGCGCGCGTCT-3′) and an antisense (5′-CCAGGATCC *TTA*CTCGTCCTCGACCATCTC GT-3′) primers, where *Nde*I and *Bam*HI-sites are underlined and the translation initiation and termination codons are italicized. PCR was performed in a 50 µl volume containing 0.2 mM each dNTPs, 2.0 mM MgCl_2_ 1.0 µM each primer, 1 µg *L. donovani* (Ag83) genomic DNA and 1.0 U Pfu DNA polymerase and 2U Taq DNA polymerase with Taq buffer (+NH_2_SO_4_). The conditions used to amplify the *LdTryS* gene was hot start at 95°C for 7 mins, denaturation at 95°C for 30 s, annealing at 60°C for 30 s, elongation at 72°C for 2.5 mins and subjected to 30 cycles with a final extension for 10 mins at 72°C. A ∼1.9 kb PCR product was observed on 1.0% agarose gel electrophoresis. This PCR product was double digested with *Nde*I and *Bam*HI, electrophoresed, purified with gel extraction kit (Qiagen), and cloned into *Nde*I and *Bam*HI- digested pET-15b in the same orientation as the T7 promoter. The ligated mixture was transformed in competent DH5α cells (Novagen) which produced the pET-15b-LdTryS plasmid. The insert and ORF orientation was confirmed by colony PCR. Construct plasmids were isolated by using Qiagen Miniprep Kit, according to manufacturer’s instruction. Two independent plasmids were sequenced to verify the gene sequence of the clones. The pET15b-LdTryS construct was transformed into competent *E. coli* BL21 (DE3) (Novagen Inc., Madison, WI) cells by heat shock at 42°C for 45 s followed by 2 mins on ice, and the cells were grown at 37°C on Luria Bertani (LB) agar medium in the presence of 50 µg/ml ampicillin (Amp).

### Expression and Purification of Recombinant LdTryS Protein

Expression of the recombinant LdTryS (rLdTryS) fusion protein in BL21 (DE3) cells was optimized to get maximum expression in the soluble fraction. The 5 ml overnight culture was used to inoculate 500 ml fresh LB-Amp medium and cultured at 37°C with shaking at 200 rpm. When the A_600_ reached between 0.5–0.6, 0.5 mM IPTG was added to induce protein expression and culture continued to grow for 24 hrs at 22°C. The *E. coli* cells were harvested by centrifugation at 5000×g for 10 mins at 4°C, washed with PBS (pH 7.2), and resuspended in 25 ml lysis buffer, (50 mM Tris-HCl pH 8.0, 300 mM NaCl, and 0.1% Triton X-100), 100 µg/ml lysozyme, and 1.0 mM phenylmethylsulfonyl fluoride (PMSF). The cell suspension was vortexed, incubated at 30°C for 30 mins, sonicated on ice and centrifuged at 14000×g for 20 mins at 4°C. The supernatant was mixed with preequilibrated 1.5 ml slurry of nickel- nitrilotriacetic acid (Ni^2+^-NTA) and incubated for 3 hrs at 4°C with gentle shaking. The resin was divided into three 10 ml disposable columns (Bio-Rad), washed with 5–8 column volumes of lysis buffer containing 10–50 mM imidazole and eluted with lysis buffer containing 100 mM, 200 mM and 300 mM imidazole, as described previously [41], [42]. The integrity and purity of the rLdTryS protein was confirmed by 10% SDS-PAGE analysis and Coomassie Brilliant Blue R-250 staining. The eluted fractions were combined and dialyzed twice against a 300 fold volume of 50 mM Tris-Cl (pH 8.0), 150 mM NaCl supplemented with 10% glycerol, overnight at 4°C.

The purified rLdTryS was digested with bovine or human thrombin (Sigma) in the column or out of the column for 6 hrs at 25°C, followed by elution and the thrombin was removed by passing the elute through a HiTrap-benzamidine column (Sigma), as per manufacturer’s instructions. The digested protein eluted from the benzamidine column was dialyzed against 100 mM PBS, pH 7.4 containing 2.0 mM DTT at 4°C [43]. The concentration of dialyzed protein was determined by Bradford method using spectrophotometer (U3900, Hitachi, Japan) and bovine serum albumin as standard [44]. The rLdTryS protein was stored at −30°C in 10% glycerol in small aliquots until use.

### Enzyme Activity of LdTryS

Enzymatic assay was performed using a continuous spectrophotometric assay at 340 nm, in which ATP is formed in a coupling reaction using an enzyme pyruvate kinase and lactate dehydrogenase and LdTryS activity is measured by monitoring oxidation of NADH to NAD^+^ at optimum pH 8.0, as described previously [18], [20]. Each 100 µl assay mixture contained 50 mM (K^+^) HEPES (pH 8.0), 0.2 mM NADH, 1.0 mM phosphoenolpyruvate, 5.0 mM TCEP, 0.5 mM EDTA, 10.0 mM MgSO_4_, 10.0 mM ATP, 10.0 mM Spd and 0.4 mM reduced GSH, 2 units per ml lactate dehydrogenase, 2 units per ml pyruvate kinase, and 5 µg of LdTryS purified protein. To determine pH optima of LdTryS the following mixed buffers were used: 50 mM 2-(*N*-morpholino)ethanesulfonic acid/NaOH for pH 5.5, 6.0, and 6.5; HEPES/NaOH for pH 7.0, 7.5, and 8.0; *N*-[tris-(hydroxymethyl)-aminomethane for pH 8.5 and 9.0; and 3-(cyclohexylamino)- 1-propanesulfonic acid for pH 9.7, 10.0, 10.5, and 11.0 as reported earlier [42]. The *K_m_* and *V_max_* values for different substrates was deduced using above mentioned reaction mixture with varying concentrations of substrates GSH (0.01–5 mM) and spermidine (0.01–10 mM) by double beam spectrophotometer (U3900, Hitachi, Japan). The data was individually fitted to the Michaelis-Menten equation and *K_m_*, *V_max_* values were calculated by Lineweaver-Burk plots. One unit of enzyme activity is defined as the amount of protein required to oxidise 1 µmol NADH to NAD^+^ in 1 min.

### Production of Polyclonal Antisera against Recombinant LdTryS and Immunoblot Analysis

Polyclonal antisera against recombinant LdTryS was raised in adult rabbit by four repeated subcutaneous injection. Pre-immune sera was collected before immunization and first dose of 250 µg LdTryS protein emulsified in complete Freund’s adjuvant was followed by three booster doses of same quantity emulsified in Freund’s incomplete adjuvant. Anti-TryS titre was checked by ELISA after three weeks of final immunization. Finally, rabbit was sacrificed, serum collected and stored at −30°C in small aliquots. Working antibodies were stored at 4°C. Prior animal ethical committee approval was taken and recommendations were strictly followed.

Total cell lysate from stationary phase promastigotes of *L. donovani* (1×10^8^ cells/ml) was prepared in PBS (pH 7.2) containing 1X protease inhibitors cocktail (Roche). The protein fractions were resolved by 10% SDS-PAGE and electro-blotted on to nitrocellulose membrane. The membrane was probed with polyclonal anti-TryS sera (1∶3000) raised in rabbit as mentioned above. ALP-conjugated goat anti-rabbit IgG (1∶2000) was used as secondary antibody and blot developed with BCIP/NBT solution (Santa Cruz), as per manufacturer’s instructions [40].

### Digitonin Fractionation of *L. donovani* Promastigotes

The differential membrane permeabilization of *L. donovani* promastigotes was done using digitonin (Calbiochem), as described previously [45], [46]. Briefly, 4×10^9^ stationary phase promastigotes (5×10^8^ cells approximately contain 1.0 mg total protein) were resuspended in 3.150 ml of HBSS buffer and aliquoted into nine fractions of 350 µl each. The harvested promastigotes were permeabilized with increasing concentration of digitonin (0–10 mg per mg of total protein). The cell suspension was incubated at 37°C for 2 mins, centrifuged at 14000×g, 4°C for 2 mins and supernatant fractions collected. Pellets were resuspended in 350 µl HBSS buffer containing 1.0% Triton X-100 and 0.5 mg/ml digitonin, incubated at 37°C for 15 mins and centrifuged at 14000×g, 4°C for 5 mins to get the pellet fractions. The supernatant and pellet fractions were resolved on 10% SDS-PAGE and analysed by immunoblot using anti-LdTryS, anti-LdIscS and anti-LdcTXN antibodies generated in our laboratory.

### Indirect Immunofluorescence Assay

*L. donovani* promastigotes in late log phase (5×10^6^ cells/ml) were labelled with 500 nM of mitochondrion specific dye, Mitotracker Red (Invitrogen) [46] in serum free M199 media and incubated for 30 mins in BOD at 24.0±1°C. The culture was harvested, washed twice with PBS, and fixed in 1.0% formaldehyde for 30 mins at RT and cells permeabilized with 0.1% Triton X-100 for 10 mins. After that, 0.1 M glycine solution was added and incubated for 10 mins at RT, centrifuged at 3000×g at RT and cell pellet resuspended in 200 µl PBS. Finally, 40 µl of the cell suspension was spread on microscopic slide and dried completely at RT. The slide was blocked with TB buffer (PBS with 0.1% Triton X-100 and 0.1% BSA). The fixed parasites were incubated with anti-LdTryS antibody diluted in TB buffer (1∶500) for 1 hr at RT and secondary antibody FITC-conjugated goat anti-rabbit IgG at 1∶2000 dilution (Santa Cruz) for 1 hr at RT. Cells were washed twice with PBS and labelled with 0.01 µg/ml DAPI (Sigma) in TB buffer for 15 mins, RT. The cells were washed thrice and immunofluorescence was observed in microscope (Model BX 41 Olympus).

### cDNA Synthesis and RT-PCR

Reverse transcription PCR was performed using 2 µg total RNA as a template isolated from *L. donovani* promastigotes of exponential and stationary growth phase, Amp B sensitive and resistant parasites and parasites treated with different concentration of H_2_O_2_ (10–200 µM). An anchored oligo(dT) primer (GenHunter; H-dT11M) was used to synthesize single strand cDNA. The semi-quantitative RT-PCR was performed using cDNA and gene specific primers (TrySF-5′-CCACCGCTACAAATCCAGTT-3′andTrySR5′-CATCGATGATGGTCCAGATG-3′) with hot start at 94°C for 5 mins, and 25 amplification cycles (94°C for 30 s, 58°C for 30 s, and 72°C for 1 min.), followed by final extension at 72°C for 5 mins. The PCR products were run on 1.2% agarose gel, stained with ethidium bromide, and finally documented and quantified using the gel documentation system and quantity one software (Bio-Rad). All the reverse transcription PCR (RT-PCR) products were normalized with respect to the α-tubulin. More than three experiments were carried out separately and consistent results were obtained. These semiquantitative data were validated by quantitative real-time PCR, which was performed in the Lightcycler 480 (Roche) using SYBER green (Roche) chemistry. The PCR parameters were 1 cycle at 95°C for 3 mins, and 40 cycles (95°C for 15 s, 58°C for 30 s, and 72°C for 30 s). The fluorescence signal was captured at the end of each cycle using the SYBER channel (490 nm wavelength for excitation and 525 nm wavelength for emission). The experiment was repeated twice in duplicate. Results were analysed by Lightcycler 480 relative quantification software (Roche) and expressed as the target/reference ratio of each sample normalized by the target/reference ratio of the calibrator.

### MTT Assay

The MTT (3-(4,5-dimethyl-2-thiazolyl)-2,5- diphenyl-2H-tetrazolium bromide) (Sigma, USA) assay is a quantitative colorimetric assay for measurement of metabolically active cells and used for determining IC_50_ value of *L. donovani* treated with H_2_O_2_. Briefly, 1×10^6^ parasites were aliquoted in each well of a 24 well plate and treated with increasing concentration of H_2_O_2_ (0–200 µM). 200 µl of cell suspension from different wells was aspirated after every 3 hours interval up to 15 hrs, mixed with 20 µl of MTT solution and incubated at 25°C for 3 hrs, as described previously by our group [47]. To solubilise the resulting formazan crystals, 200 µl of MTT solubilisation buffer was added and optical density of the solution measured at 570 nm. The percentage of cell viability was determined by comparing to untreated *L. donovani* cultures. The experiment was performed three times in duplicate.

### Quantitation of Reactive Oxygen Species

Fluorescence spectrophotometry was used to measure the production of intracellular reactive oxygen species (ROS) using 2′–7′-dichlorodihydrofluorescene diacetate (H_2_DCFDA) (Sigma) as a probe, which is oxidized inside the cell to the fluorescent dichlorofluorescein, as described previously [47]. Briefly, *L. donovani* promastigotes were treated with different H_2_O_2_ concentrations (10 µM, 25 µM, 50 µM, 100 µM, 150 µM and 200 µM) for 8 hrs in a 24 well plate. Cells were washed with PBS and 2×10^6^ cells were incubated in 1.0 ml of PBS containing 50 µM H_2_DCFDA for 30 mins in dark. Total 1×10^6^ cells were taken from each well, washed once in PBS, lysed in lysis buffer (1% SDS and 1% Triton X-100 in 10 mM Tris-HCl, pH 8.0) and the fluorescence intensity was immediately read using LS55 spectrofluorometer (Perkin Elmer), with excitation measured at 492 nm and emission at 529 nm. The measured fluorescence is directly proportional to the accumulation of ROS and expressed in relative fluorescence unit (RFU). The reagent blank was prepared with 50 µM H_2_DCFDA in lysis buffer. The accumulation of ROS has also been measured in the presence of ROS quencher *N*-acetyl-L-cysteine (Amresco). Untreated parasites were used as control. The experiments were performed three times independently in duplicate.

### Analysis of LdTryS Expression in Oxidative Stress

*L. donovani* promastigotes in late log phase were treated with different concentration of H_2_O_2_ from 10 µM to 200 µM for 8 hrs. LdTryS expression level was measured in harvested parasites. Briefly, culture was harvested and washed twice with PBS. The cell pellets were resuspended in PBS containing protease inhibitor (cocktails), lysed with 3 freeze thaw cycles, sonicated and centrifuged at 12000×g, 4°C for 20 mins. Immunoblot analysis was carried out using anti-LdTryS (1∶3000) and anti-β-actin (1∶2000) followed by ALP conjugated goat anti-rabbit IgG (1∶2000). Real time quantitative PCR analysis was conducted from total RNA isolated from untreated and H_2_O_2_ treated parasites, as described above. The experiment was performed twice independently in duplicate.

## Results

### Cloning, Expression and Purification of LdTryS Protein

**Figure 1:**
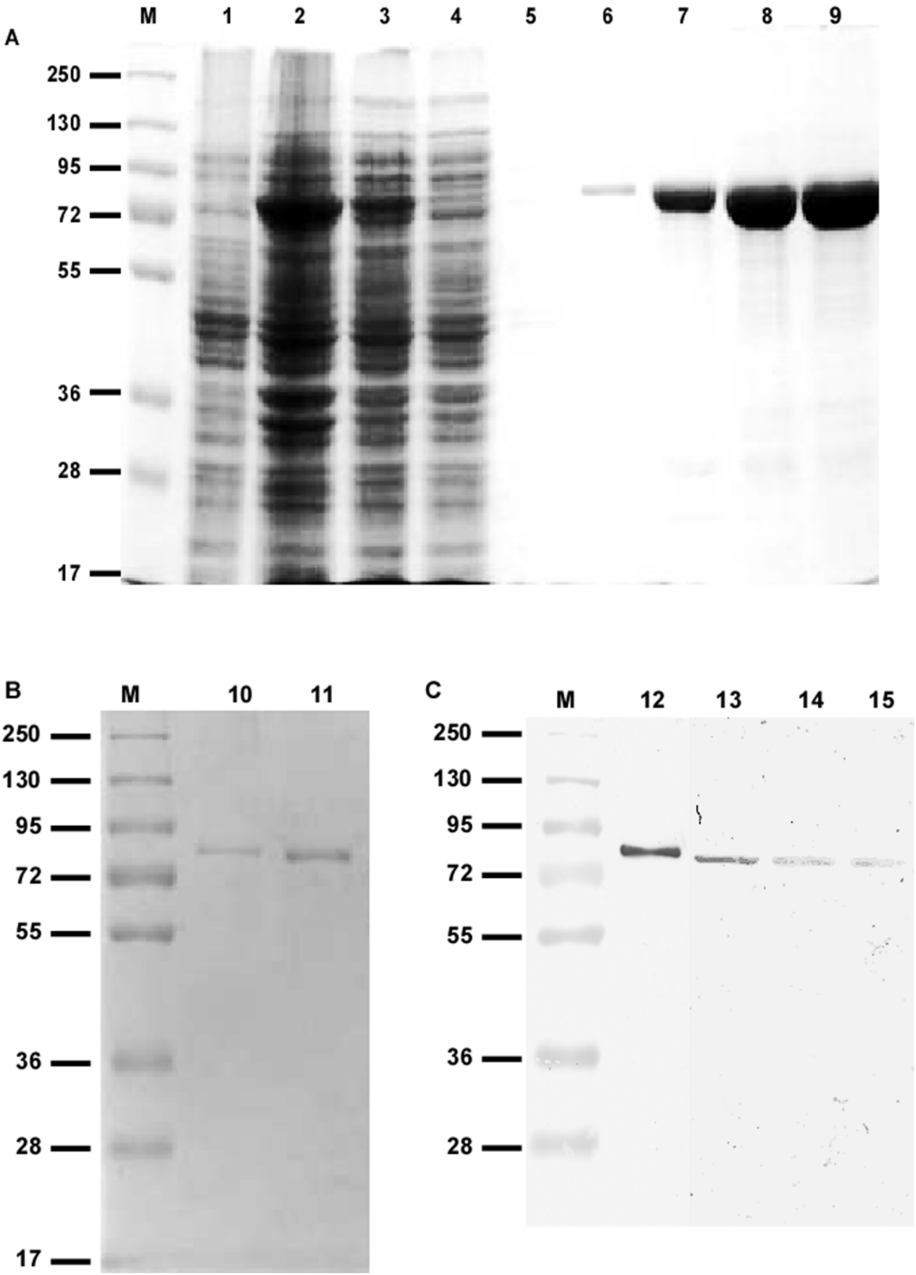
Expression and purification of recombinant LdTryS protein. (A): LdTryS was expressed as a fusion protein containing the amino-terminal histidine tag and purified with the Ni^2+^-NTA column as described in “Materials and Methods”. The total cell lysate and samples at each purification step were electrophoresed on 10% SDS-PAGE gel and stained with coomassie brilliant blue. Lane M, protein marker; lane 1, an *E. coli* transformant with pET-15b empty vector as control; lane 2, total lysate of cells expressing LdTryS gene; lane 3, supernatant of lane 2 after centrifugation at 21,000 rpm; lane 4, unbound fraction of the Ni^2+^-NTA column; lane 5, 6 wash from the Ni^2+^-NTA column with 10, 50 mM imidazole; lane 7, 8 and 9 elutes from the Ni^2+^-NTA column with 100 mM, 200 mM and 300 mM imidazole, respectively. (B): Coomassie staining of undigested and thrombin digested rLdTryS protein. Lane 10, undigested rLdTryS; lane 11, thrombin digested rLdTryS. (C): Western blot with anti-LdTryS sera. Lane 12, represents 10 ng of rLdTryS protein and lane 13, represent 20 µg total cell lysates of *L. donovani*, lane 14 and 15, represent 20 µg supernatant and pellet fractions, respectively.

The *TryS* gene was amplified from genomic DNA of *L. donovani* and the resulting 1959-bp fragment was cloned into pET-15b giving the plasmid pET15b-LdTryS. The *LdTryS* ORF 1959 nucleotides encodes a protein 652 amino acids with predicted molecular weight ∼74 kDa and an iso-electric point (pI) value 5.57. Neither the MITO-PROT II program nor Signal IP 4.1 Server, which predicts protein localization in cells, and a Kite-Doolittle hydropathy plot, suggested any possible cellular localization other than cytosolic distribution for LdTryS. The recombinant LdTryS was expressed in *E. coli* BL21 (DE3) and purified to homogeneity using Ni^2+^-NTA affinity chromatography as shown in Fig. 1A. It was observed that LdTryS expression was higher in soluble form and eluted between 100–300 mM imidazole with high protein yield of ∼8.0 mg/ml. The purified rLdTryS protein gave a single band of 76 kDa when examined on SDS-PAGE and immunoblot using anti-histidine monoclonal antibody which correlates well with predicted molecular mass 74 kDa and behaves as a monomer on gel filtration (Data not shown). The (His)_6_-tag was removed by thrombin, as described in materials and methods, to achieve complete protein without (His)_6_-tag for further work. Online tool peptide cutter suggested that thrombin may digest at position 313 a. a. corresponding to Pro-Arg-Ile sequence. However, both bovine and human thrombin did not cleave at position 313 which suggests that thrombin practically recognise and cleave only Pro-Arg-Gly sequence, as shown in Fig. 1B. The undigested rLdTryS was observed at a slightly higher molecular weight due to the presence of a 2.6 kDa (His)_6_-tag at N-terminal. However, digested rLdTryS protein is similar in size to the predicted molecular mass confirming the molecular mass of native *L. donovani* protein.

Polyclonal antisera against recombinant purified rLdTryS was raised in adult rabbit and titre measured at final bleed was high (1∶16000 dilution). Antibody against rLdTryS recognised a specific and single band both in *L. donovani* lysate protein and purified rLdTryS, suggesting that a single homologue is present in the parasite (Fig. 1C). It was further observed that although majority of protein was present in the soluble fraction, but a trace amount was also detected in the pellet fraction indicating that a small amount of protein may be associated with membrane fraction. This was further confirmed by localization studies (discussed later) that majority of LdTryS protein, if not all, is present in the cytoplasm. By immunoblot of a series of diluted recombinant proteins (data not shown), we roughly estimated that the *Leishmania* contains a significant amount of LdTryS, which consists of approximately 0.01% of the total protein.

### Features of LdTryS Protein and Sequence Alignment

**Figure 2:**
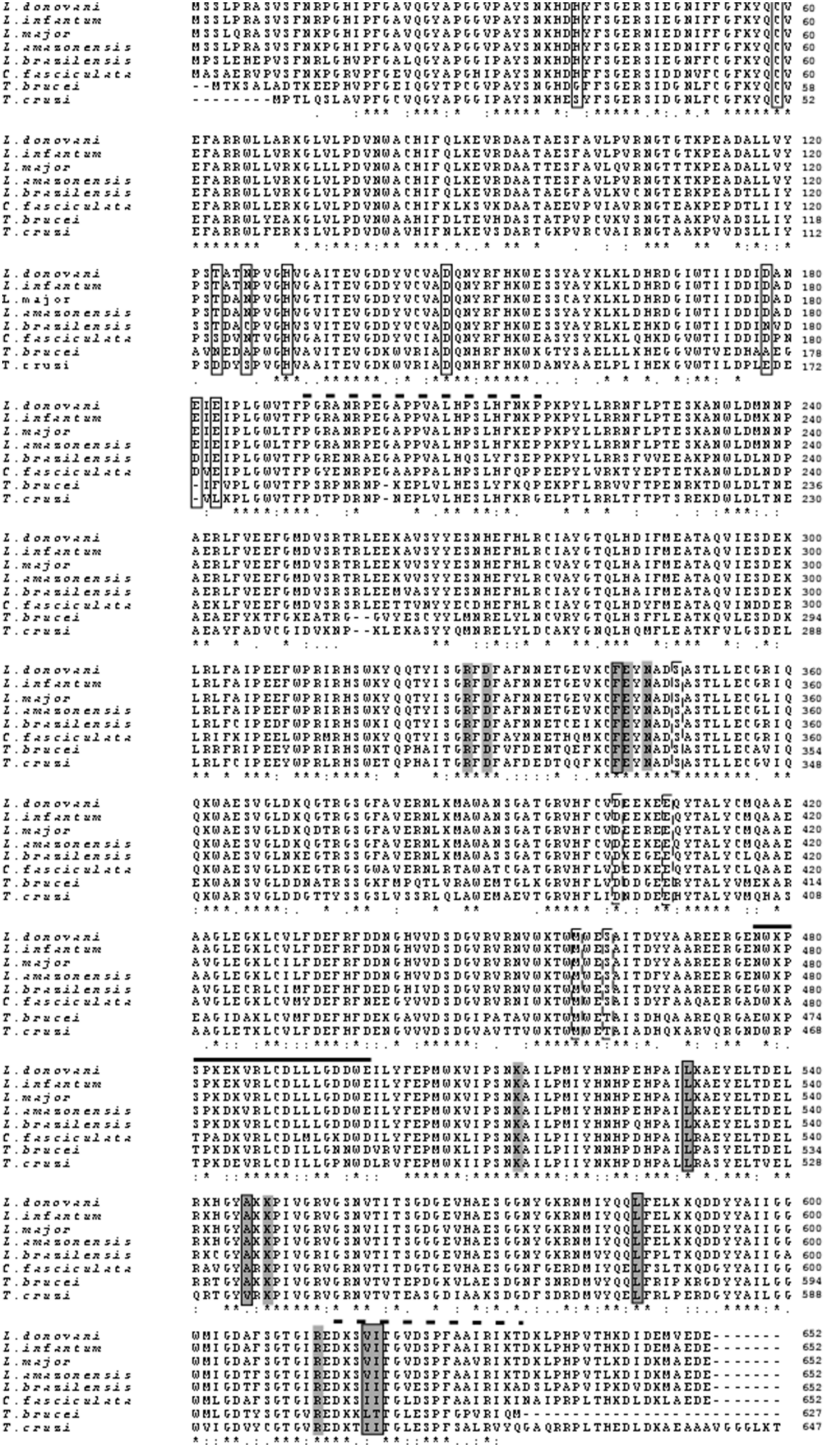
Multiple sequence alignments of deduced amino acid sequences of TryS from *L. donovani* and other organisms. Protein sequences were aligned using the CLUSTAL W program (www.ebi.ac.uk/clustalw/). Sequences are *L. donovani* (CAD23679), *L. infantum* (XP_001466426), *L. major* (XP_003721994), *L. amazonensis* (ABQ57409), *L. braziliensis* (XP_001565955), *C. fasciculata* (AAT99012), *T. brucei* (CAC87573), and *T. cruzi* (XP_816076). *Asterisks* indicate identical amino acids. *Dots* and *colons* indicate conserved amino acid substitutions. *Dashes* indicate gaps. *Closed boxes* at the amino terminus indicate conserved amino acids involved in amidase activity in all *Leishmania* species except *L. braziliensis*. *Closed dashed boxes* at the central region (on the 6^th^, 7^th^, and 8^th^ rows) interact with GSH. *Gray boxes* at the central region and C-terminal region indicate amino acids involved in synthetase activity and *gray closed* boxes show a. a. involved in binding triphosphate moiety of ATP. *Dashed lines* above the alignment indicate the linker regions between amidase and synthetase domain at N- and C-terminal region of the protein. A *Solid line* above the alignment indicates unique insertion in TryS of eukaryotes which is absent in prokaryotes TryS.

The alignment of 14 TryS/GSPS homologues from *Leishmania*, *Trypanosoma*, and bacteria revealed (an alignment of only representative members is shown in Fig. 2) that LdTryS showed highest nucleotides homology, (99–94%), to TryS from *L. infantum*, *L. amazonensis,* and *L. major.* Genome database analysis of *L. donovani* revealed the presence of single TryS gene with high amino acids identities (99 to 78%) to *L. infantum*, *L. amazonensis, L. major, L. braziliensis, C. fasciculata,* moderate identity (∼60%) with *T. brucei, T. cruzi* TryS and very low a. a. identities with *E. coli* TryS (26%) and *E. coli* GSPS (31%). LdTryS protein contains amidase activity at N-terminal (1–215 a. a.) and C-terminal region (634–652 a. a.), whereas, synthetase activity is present in the central region ∼400 a. a. and this region is attached with linker to the N-terminal and C-terminal amidase domain, as described in *L. major* [24]. The linker segment between N-terminal amidase to synthetase and synthetase to C-terminal amidase correspond to 191–216 a. a. and 615–633 a. a., respectively, as shown by dashed line above the alignment. The synthetase active site is a triangular shaped cavity which forms three substrate binding domains that can accommodate each substrate; i.e., ATP, GSH, and Spd between their clefts. Based on the crystal structure analysis of LmTryS, the adenine of ATP molecule binds to the conserved residues Phe-343, Leu-530, Ala-546, Leu-585, Val-618, and Ile-619 in LdTryS, as shown in gray closed boxes (Fig. 2). Similarly, triphosphate moiety of ATP also binds to the conserved Arg-328, Asp-330, Glu-344, Asn-346, Lys-513, Lys-548 and Arg-613, as shown in gray boxes (Fig. 2). The second substrate, GSH, forms hydrogen bonds and Van der-Waal interactions with Ser-349, Asp-403, Glu-408, Met-459, and Ser-462 as shown by closed dashed boxes, similar to LmTryS. The Ser-462 of LdTryS is substituted by Thr-456 in *T. brucei* and Thr-450 in *T. cruzi* which may not affect the GSH binding due to replacement by polar hydrophobic a. a. with similar chemical properties. The third substrate, Spd or Gsp, binds to the pocket formed by Ser-351, Glu-355, & Glu-407 of LdTryS conserved in other *Leishmania* species (Fig. 2) but conformational changes occur during enzyme substrate interaction [24].

The amidase active site is present mainly at N-terminal domain and major a. a. involved in catalysis are His-39, Cys-59, His-130, and Asp-146 conserved in all *Leishmania* species, as shown in closed boxes (Fig. 2). Site-directed mutagenesis identified Cys-59 as a catalytic cysteine [18] and this catalytic triad is completed by His-130 and Asp-146. At the C-terminal, three acidic amino acids (Glu-650, Asp-651, and Glu-652) participate in hydrogen bonding and salt bridging interaction with basic a. a. side chains and in turn, blocks access to the catalytic Cys-59 responsible for amidase activity. The C-terminal Glu-652 interacts with Arg-383, and His-39 and the side chain accept hydrogen bonds donated by Cys-59 and Asn-148. It was further observed that Spd or Gsp are directed out of the active site towards an acidic cleft and conserved residues Thr-123, Asp-178, Glu-181, and Glu-183 interacts with the substrate, as shown by closed boxes (Fig. 2). Near the catalytic site, His-39 and Asn-126 may interact with the central amine of Spd [24]. A bold line above the alignment in Fig. 2, from 476–498 amino acids, is a unique insertion in eukaryotes TryS which is missing in bacterial homologues. However, this insertion is also present in *E. coli* GSPS with very low amino acids identity whereas it showed 100% a. a. identities in all *Leishmania* species except *L. braziliensis* (90%) in which Asn-476 and Glu-498 are substituted with Gly-476 & Asp-498, respectively. It was also observed that amino acids 487–494 form a α-helix and other amino acids produce a loop-like structure containing two exposed tryptophan which may play role in conformational changes that occur during Gsp binding to synthesize T(SH)_2_.

### Enzymatic Activity and Multimeric Structure

**Figure 3:**
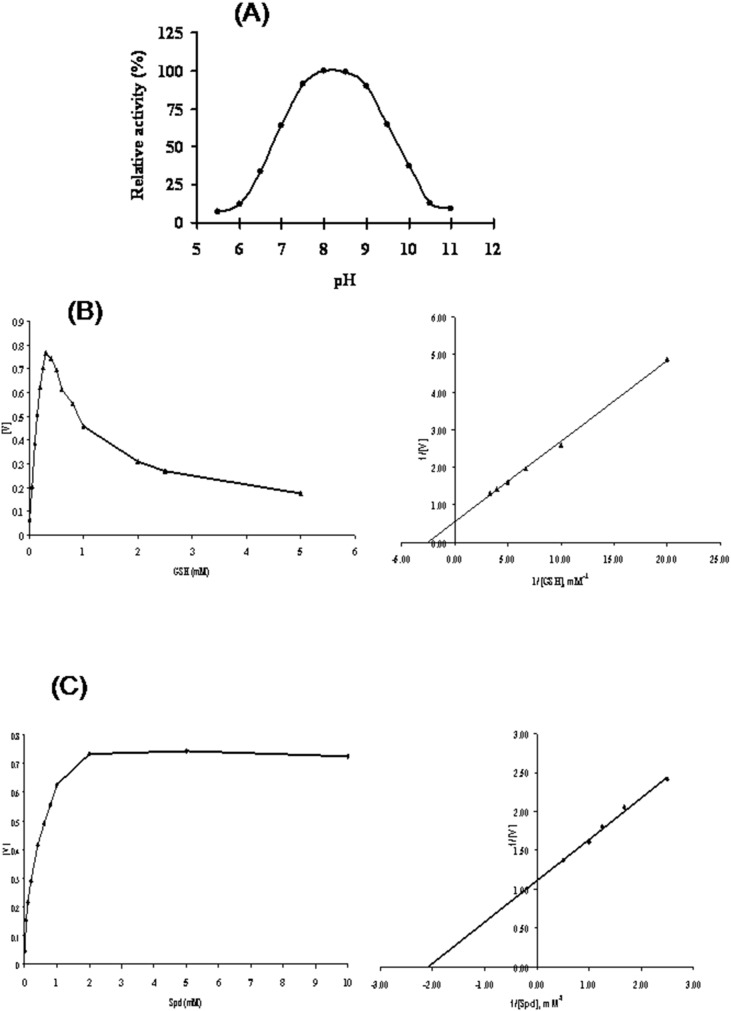
Enzymatic analysis of recombinant purified LdTryS. (A) pH profile using a coupled assay in a mixed buffer system. Activity is expressed as a percentage relative to the maximum activity observed with LdTryS. Kinetic properties of LdTryS with substrates GSH (B) and Spd (C) were analysed. *K*_m_ values were determined for each substrate by fitting data into Michaelis Menten equation and resulting Lineweaver-Burk plots. The experiments were performed three times in duplicate and data presents the mean ± SD.

The purified rLdTryS enzyme showed a concentration dependent TryS activity in 50 mM HEPES buffer, pH 8.0 and deduced specific activity for GSH and Spd were found to be 2.94±0.15 and 6.688±0.23 µmoles/min/mg of purified LdTryS protein, respectively. The optimum pH for rLdTryS activity was found to be 8.0–8.5 (Fig. 3A) which is similar to previous reports for *L. major*, *T. cruzi* [18], [20], *L. donovani* [22] & *T. brucei* TryS [5]. The LdTryS activity gradually decreased at higher or lower pH. The LdTryS showed substrate inhibition, especially GSH, over 0.2 mM which is similar to that observed in other trypanosomatids. Further, the affinity *K*_m_ for substrates, GSH & Spd, was found to be 0.37±0.03 and 0.475±0.01 mM, respectively (Fig. 3B & 3C), which is higher than earlier reported for *L. donovani* [22] and *L. major* [20], *T. brucei* [5] but lower than *T. cruzi* [18]. The *V*_max_ values for GSH and Spd of LdTryS was found to be 2.08±0.085 and 0.915±0.083, respectively calculated by Lineweaver-Bulk plots.

To determine multimeric structure of LdTryS, the purified rLdTryS protein (∼500 µg) was dialyzed against 100 mM Tris-HCl, pH 8.0, 100 mM NaCl buffer O/N at 4°C, concentrated up to 1.0 ml and applied on Sephacryl S-300 column (50×0.7 cm) pre-equilibrated with standard protein marker (Amersham BioSciences). Column was run at a flow rate of 0.5 ml/min. The rLdTryS eluted before albumin at the predicted molecular weight 75–80 kDa, which proves that rLdTryS exists as monomer (data not shown), as reported previously for all other organisms.

### Localization of LdTryS

**Figure 4:**
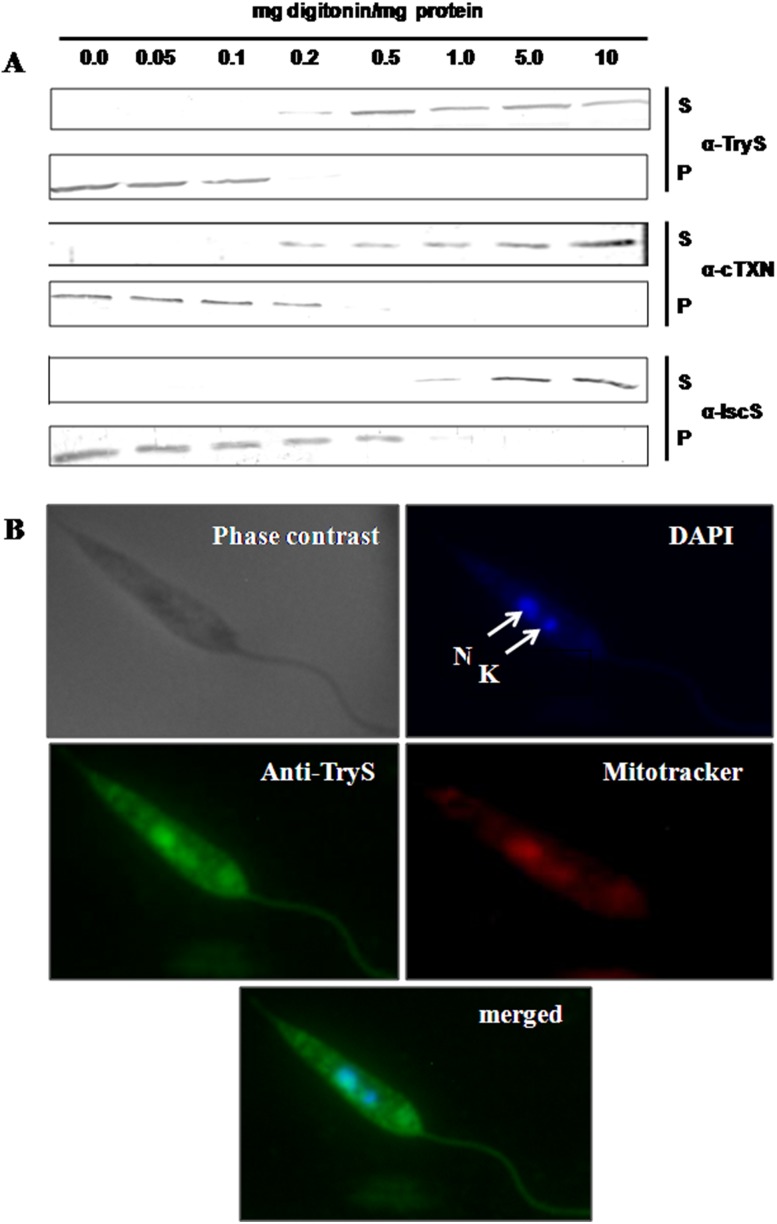
Subcellular localization of LdTryS. (A) Differential digitonin permeabilization of stationary phase promastigotes with increasing concentrations of digitonin. Supernatant and pellet fractions were run on 10% SDS-PAGE and transferred on to nitrocellulose membrane for western blot analysis using anti-LdTryS (1∶3000), anti LdcTXN (1∶4000), and anti LdIscS (1∶2000). cTXN and IscS served as cytosolic and mitochondrial markers, respectively. (B) Immunofluorescence microscopy of *L. donovani* promastigote with anti-LdTryS sera: phase contrast image, DAPI stained nucleus (N) and kinetoplast (K), Mitotracker stained mitochondria, anti-TryS labeled promastigote along with its merged image with DAPI is showing TryS localization in the cytoplasm.

The subcellular localization of LdTryS was analysed by differential digitonin permeabilization of parasites, as permeabilization of the cell depends on the cholesterol content of membranes [48]. LdTryS is released from cells at relatively low digitonin concentration (above 0.1 mg digitonin per mg total protein), similar to LdcTXN, whereas, mitochondrial protein LdIscS (unpublished data) starts to release at 0.5 mg digitonin per mg total protein as shown in Fig. 4A. It was observed by analysis of the pellet fractions that higher concentrations of digitonin (over 1.0 mg per mg total protein) is required to completely release LdIscS whereas cytosolic proteins are completely released up to 0.2 mg digitonin. Since LdTryS release pattern is similar to the cytosolic marker LdcTXN and differs significantly from mitochondrial control LdIscS, we can conclude that LdTryS is localized in the cytosol.

The cellular localization of TryS in *L. donovani* promastigotes was further confirmed by Indirect Immunofluorescence Assay (IFA), as described in materials and methods. Immunofluorescence staining of *L. donovani* promastigotes with anti-LdTryS polyclonal antibody showed extensive and even staining throughout the cells, except for the nucleus and kinetoplast (Fig. 4B). The nucleus and kinetoplast were labelled by DAPI, and mitochondria labelled by mitotracker. The merged image of DAPI and anti-LdTryS labelled image did not overlapped and a comparison with mitotracker labelled image suggests cytosolic/non-organellar localization of LdTryS. It was previously reported in *L. major* that TryS is predominantly found in the cytosol [20]. Similarly, our results based on subcellular digitonin fractionation and immunofluorescence microscopy revealed that TryS is predominantly found in the cytosol of *L. donovani*. MITO-PROT II and Target-P analysis of LdTryS showed the absence of any targeting sequence which again supports its cytosolic localization.

### Analysis of LdTryS Expression in Exponential Vs. Stationary Phase

**Figure 5:**
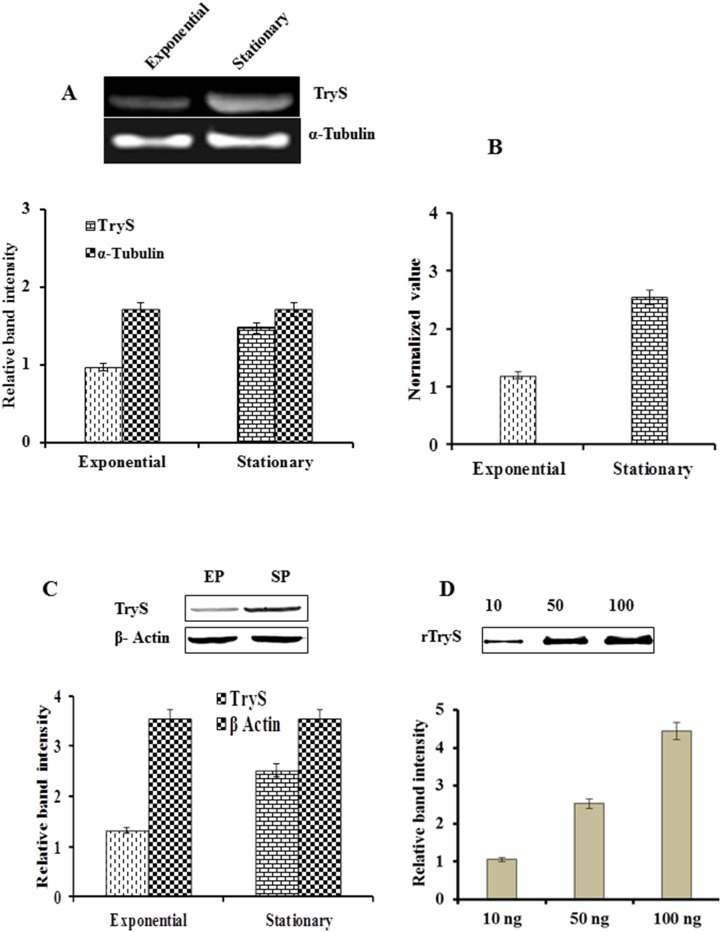
Determination of expression level of LdTryS in exponential vs. stationary phase of *L. donovani.* (A) Semiquantitative RT-PCR analysis of LdTryS transcript in exponential and stationary phase promastigotes. Ethidium bromide-stained PCR products were photographed and the image was analyzed densitometrically. α-tubulin was used as control to show uniform expression of a housekeeping gene in both stages of promastigotes. (B) Bar graph represents quantitative real time PCR analysis of LdTryS expression level in exponential vs. stationary phase promastigotes. Data are normalized by the target/reference ratio of the calibrator. (C) Western blot of 30 µg total *Leishmania* lysate proteins from exponential phase (EP) and stationary phase (SP), and image was analyzed densitometrically. Data was normalized and β-actin was used as control. (D) Western blot of increasing quantities of recombinant LdTryS used as standard and analyzed densitometrically to compare the expression level of TryS in *Leishmania* lysate. The experiments were repeated thrice and data represents the mean ± SD.

It was earlier reported that expression of tryparedoxin is ∼15-fold upregulated in axenic amastigotes of *L. infantum* [49]. The expression of this protein also differs between exponential and stationary phase promastigotes. The stationary phase or metacyclic promastigotes are infective stage of parasites in contrast to exponential growth phase promastigotes. The microarray data has shown that TryS mRNA level is 1.8-fold higher in promastigotes as compare to axenic amastigotes of *L. infantum* (http://tritrypdp.org). Keeping this point in mind, we tried to check expression of LdTryS in exponential Vs stationary phase of *L. donovani* promastigotes at transcriptional as well as translational level. The expression pattern of LdTryS transcript was analysed by semiquantitative and real time PCR in both exponential and stationary phase promastigotes. The intensity of band signals was ∼2-fold higher in stationary phase, suggesting that LdTryS is constitutively over transcribed in this phase (Fig. 5A). The above data was validated by real time PCR, and similar variations were observed in the expression level of the *LdTryS* gene (Fig. 5B). The expression level of LdTryS protein in both developmental stages was also checked and a 1.8-fold higher expression was observed in stationary phase promastigotes (Fig. 5C). A known amount of rLdTryS protein was used as control for densitometric analysis (Fig. 5D). Therefore, we can say that the expression of LdTryS is stage-dependent and higher in stationary phase promastigotes of *L. donovani* (∼2.4-fold at transcriptional level and 1.8-fold at translational level).

### Variation in Expression of LdTryS Protein in Amp B Sensitive Vs. Resistant Isolates of *L. donovani*

**Figure 6:**
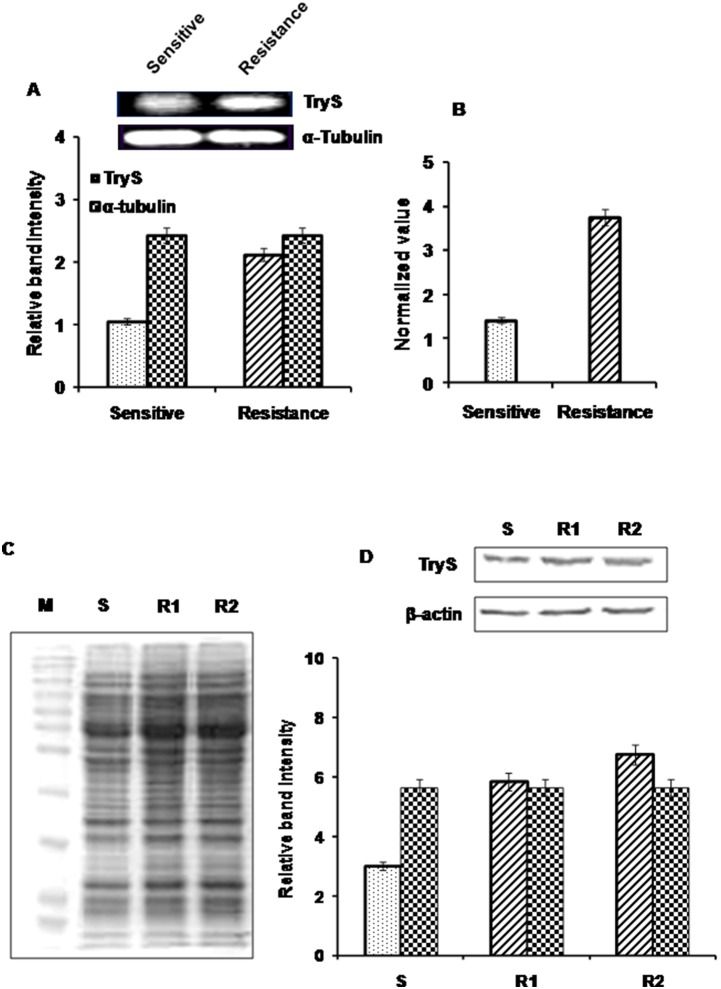
Determination of expression level of LdTryS in sensitive (S) vs. drug resistant (R) strains of *L. donovani.* (A) Semiquantitative RT-PCR analysis of LdTryS transcript in Amp B sensitive vs. resistant isolates. Ethidium bromide-stained PCR products were photographed and the image was analyzed densitometrically. α-tubulin was used as control to show uniform expression of a housekeeping gene in both Amp B sensitive and resistant promastigotes. (B) Bar graph represents quantitative real time PCR analysis of LdTryS expression level in Amp B sensitive vs. resistant isolates. Data are normalized by the target/reference ratio of the calibrator. (C) The total *Leishmania* lysates (30 µg) were electrophoresed on 10% SDS-PAGE gel and stained with coomassie brilliant blue. Lane 1 represents, protein marker; lane 2 represents, sensitive strain (S); lanes 3, and 4 represent, resistant isolates (R1, & R2). (D) Shows western blot of same coomassie gel using anti-LdTryS (1∶3000). The image was analyzed by densitometrically. Data was normalized and β-actin was used as control. The experiments were repeated twice and graphs represent the mean ± SD.

It was reported earlier that thiol level is up-regulated in SAG resistant *L. donovani* isolates due to increased expression of γ-GCS & ODC, both involved in T(SH)_2_ biosynthesis [50]. The *Leishmania* ABC transporter p-glycoprotein A (pgpA) is also involved in metal resistance [51], but exact mechanism by which it confers resistance to antimonials remains unknown. It was also earlier reported that TryS mRNA expression increased in hydroxylurea resistant *L. amazonensis* along with other genes, i.e., *ODC, TryR, TXN*, & *TXNPx* [15] and similar observation was also reported from another group on Amp B resistant *L. donovani* [39]. The Amp B resistance has been shown to be associated with higher T(SH)_2_ and TryS mRNA levels but its correlation with LdTryS protein has not been elucidated. So, we investigated the variations in LdTryS expression at mRNA and protein level of Amp B sensitive and resistant strains. Semiquantitative analysis of LdTryS mRNA showed that the gene was over transcribed in Amp B resistant strains as compared to sensitive strains (Fig. 6A). This result was further validated through real time PCR and found that *LdTryS* gene was transcribed ∼3-fold higher in Amp B resistant isolates (Fig. 6B). Similar variation was observed at protein level by immunoblot analysis where LdTryS expression was ∼2.0 fold higher in Amp B resistant isolates (Fig. 6D). The coomassie staining of same gel is shown in Fig. 6C and β-actin protein was used as loading control (Fig. 6D) [52]. Our results demonstrate that LdTryS is up-regulated in Amp B resistant clinical isolates (∼3-fold at transcriptional level and 2-fold at translational level) which is in accordance with previous findings that enzymes of thiol metabolism are upregulated in laboratory induced drug resistant parasites [15], [39]. Thus, up-regulation of LdTryS correlates well with Amp B resistant isolates which show 7–8 fold higher EC_50_ values) for Amp B (Fig. S1) as mentioned in material and methods.

### H_2_O_2_ Mediated ROS Generation and its Effect on Parasite Survival

**Figure 7:**
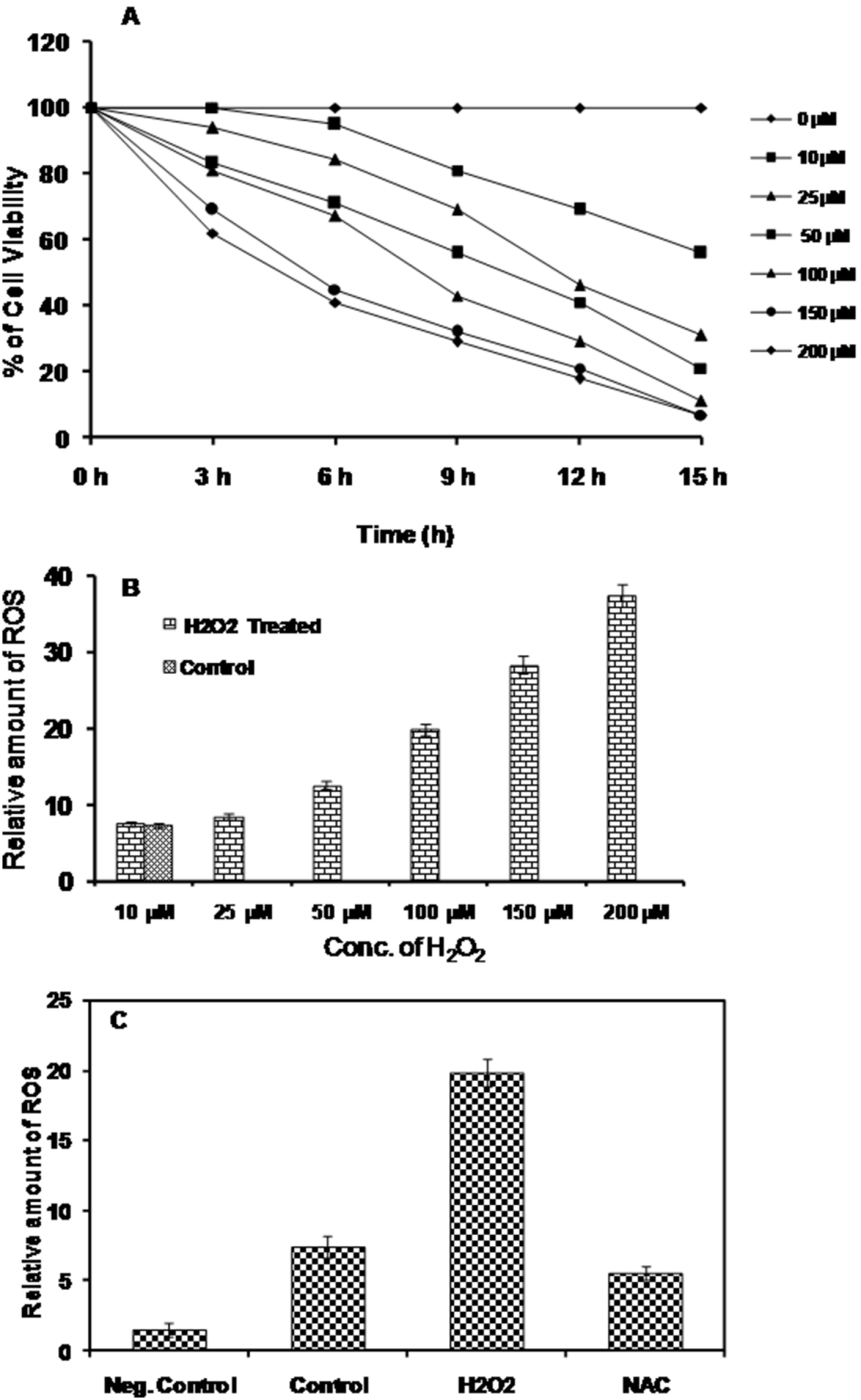
Effect of H_2_O_2_ on growth inhibition of *L. donovani* parasites. (A) *L. donovani* promastigotes (1×10^6^ cells/ml) culture was treated with increasing concentration of H_2_O_2_ (0–200 µM) up to 15 hrs and growth inhibitory effect of H_2_O_2_ determined by MTT assay at 3 hr intervals. The cell viability after exposure with increasing concentration of H_2_O_2_ was determined to optimize time of exposure and dose. (B) The intracellular ROS level was determined by quantification of DCF fluorescence. Results were normalized with cell numbers and presented relative to untreated control cells. (C) To confirm intracellular ROS production a quenching study was performed. The parasites treated with H_2_O_2_ in the presence of 20 µM *N*-acetyl-L-cysteine (NAC) ROS scavenger reversed the effect of H_2_O_2._ The experiments were repeated three times and graphs represent the mean ± SD.

The intracellular ROS production after H_2_O_2_ treatment and its effect on cell viability was measured in a time and concentration dependent manner using H_2_DCFDA. The cell viability decreased in a time and concentration dependent manner and after 15 hrs of 100–200 µM H_2_O_2_ treatment, cell viability was below 10%, as shown in Fig. 7A. It was observed that 50% cell viability remained at 50–150 µM H_2_O_2_ concentrations between 5–10.5 hrs. Thus, we selected 100 µM H_2_O_2_ for 8 hrs as optimum treatment for further analysis. For the H_2_O_2_ treatment study, we incubated the parasites with various concentration of H_2_O_2_ for 8 hrs and observed that in stress-induced parasites, ROS level was unchanged up to 25 µM H_2_O_2_ concentration. The ROS content started to increase from 25 µM H_2_O_2_ and was 5-fold higher at 200 µM H_2_O_2_ compared to untreated *Leishmania* parasites, as shown in Fig. 7B. To know whether this ROS generation is due to H_2_O_2_ treatment of the parasites or produced by the parasites, we performed a quenching study. Treatment with 20 µM *N*-acetyl-L-cysteine (ROS quencher) significantly decreased the ROS level in the stressed parasite compared to untreated, Fig. 7C. This confirmed that *in vitro* generation of ROS is directly contributed by H_2_O_2_ treatment.

### ROS Dependent Up-regulation of LdTryS Expression

**Figure 8:**
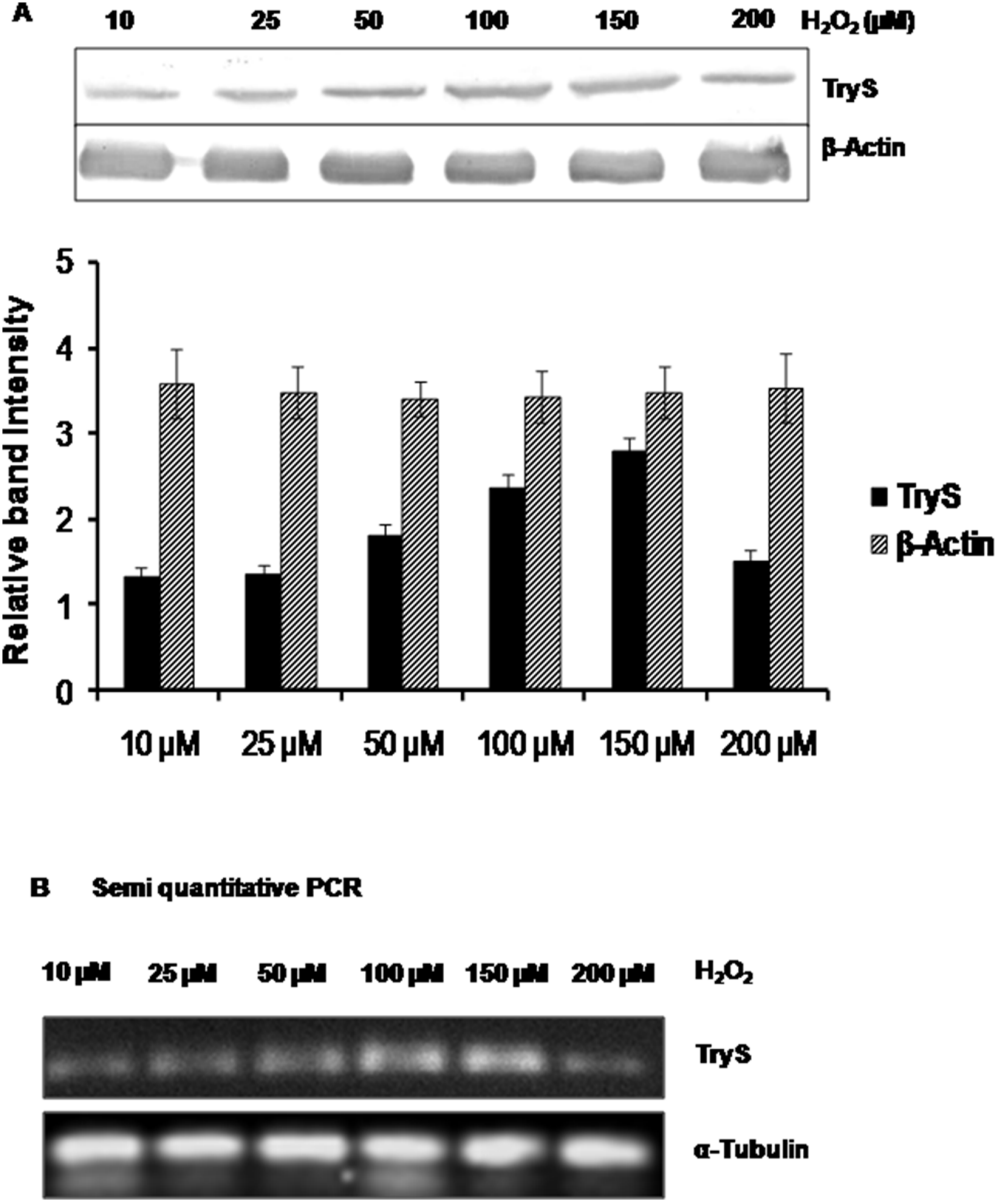
Up regulation of LdTryS in *L. donovani* promastigotes in response to H_2_O_2_ treatment. (A) TryS expression in *L. donovani* parasites in the presence of H_2_O_2_ (10–200 µM) was analysed by western blot. H_2_O_2_ treated parasites showed increased expression level of LdTryS, whereas the β-actin expression level did not change significantly. The experiments were repeated twice in duplicates and quantitation was done by densitometric analysis using Quantity One (Bio-Rad). Band intensity is presented as fold increase/decrease of LdTryS expression. (B) LdTryS expression level was analyzed by semiquantitative RT-PCR, and PCR product stained with ethidium bromide and photographed. PCR of α-tubulin was used as housekeeping control that showed uniform expression pattern irrespective of H_2_O_2_ concentration.

The *Leishmania* parasites have to encounter oxidative stress during their survival in the macrophages and during drug exposure (Amp B). To know whether LdTryS expression depends on ROS generation or it plays any role in overcoming oxidative stress condition, we checked the expression of LdTryS in the parasites exposed to various concentration of H_2_O_2_ by immunoblot. The expression of LdTryS increased up to 150 µM and then surprisingly decreased at 200 µM H_2_O_2_. No change in the level of housekeeping control β-actin compared with LdTryS was observed as shown in Fig. 8A (upper panel) and densitometric analysis of western blot, shown in Fig. 8A (lower panel). Further, semi-quantitative RT-PCR analysis of *LdTryS* also showed that *LdTryS* gene is transcriptionally upregulated from 50 to 150 µM H_2_O_2_ as compared to housekeeping control α- tubulin, Fig. 8B. Thus, the results indicate that LdTryS is induced during oxidative stress condition which may help parasites survival in the macrophages or insect gut.

## Discussion

The trypanosomatids lack glutathione reductase (GR), thioredoxin reductase (TrxR), glutathione peroxidase (Gpx), and catalase and their major thiol is synthesized by GSPS/TryS which maintain redox potential essential for the parasites survival. T(SH)_2_ is synthesized by a single TryS gene in *L. donovani*, which is absent in their mammalian host. The LdTryS is upregulated in stationary phase and Amp B resistant parasites, which is positively modulated in response to H_2_O_2_ treatment in sensitive strains indicating its role in overcoming oxidative stress encountered by the parasites during infection. It is already known that stationary phase of promastigotes is the infective metacyclic stage where the parasite undergoes significant metabolic changes. This metabolic shift involves alteration in the expression level of many proteins. T(SH)_2_ is the major thiol involved in redox homeostasis and may have greater utilisation during this stage. The intracellular ROS production is higher in late stages of growth that may contribute to enhanced T(SH)_2_ utilisation or direct TryS overexpression during this stage. Nevertheless, since LdTryS is the only gene responsible for T(SH)_2_ biosynthesis in *L. donovani*, it is overexpressed to maintain redox potential. In the present study, we found that the expression of LdTryS mRNA as well as protein is upregulated at stationary phase as compared to exponential phase (Fig. 5A, 5C).

The TryS contains synthetase domain which synthesizes T(SH)_2_ and amidase domain which regulates T(SH)_2_ concentration in cells, both functions in opposite direction. It is very interesting that a single fused protein carries out both functions very nicely to maintain homeostasis and works at different pH serving as an adaptation for parasites biphasic life cycle, macrophages and insect (sandfly gut). The existence of opposite activities in the same enzyme appears counterintuitive since this enzyme maintains a futile cycle of synthesis and degradation of the products with the expense of ATP. The optimum pH reported for amidase and synthetase activity is 6.8 and 8.5, respectively [53]. Thus, a plausible explanation of the metabolic activity of TryS may lie on pH dependent regulation of the amidase/synthetase activity, as reported for *E. coli* Gsps [53]. We also observed maximum rLdTryS activity at pH 8.5, whereas, the *K*_m_ values were slightly higher for both the substrates, GSH & Spd, as compared to *L. major* [18], *L. donovani* [22] and *T. brucei* [5] indicating a lower substrate affinity. However, differences in apparent *K*_m_ values cannot be used to assess its physiological significance in *Leishmania* parasites. Also, in *E. coli* and *C. fasciculata*, the content of Gsp, GSH, and Spd has been shown to be growth phase dependent and Gsp level increases while GSH, Spd decreases during stationary phase [54], [55] to increase biosynthesis of T(SH)_2_. The biphasic life cycle of the parasites encounter different pH which may likely shift enzymatic activities of TryS. As optimum pH for synthetase activity is basic, it may correlate the promastigotes stage. Similarly, lower pH (5.5) may lead to higher protonation and hence high amidase activity and low synthetase activity in amastigotes stage, in contrast to promastigotes. In addition, TryS expression is 1.8-fold higher in promastigotes based on microarray as compare to axenic amastigotes of *L. infantum* (http://tritrypdp.org/tritrypdp) suggesting that TryS expression is stage-specific and depends on the environmental pH of the host or vector.

Parasites redox biology has been frequently implicated in SAG as well as Amp B resistance in *Leishmania*. In laboratory-induced, SbIII selected resistant parasites have shown elevated levels of T(SH)_2_ as compare to sensitive parasites [51] whereas some resistant parasites display an associated amplification of pgpa (p-glycoprotein-like protein A) gene to facilitate the efflux of metal thiol conjugates [27], [56]. The mechanism of drug resistance was known as active efflux of the drug, but parasites redox biology is also implicated in drug resistance. Similarly, SAG resistant *Leishmania* isolates have been reported to have increased reserve level of T(SH)_2_ responsible for SAG resistance [57]. Also, up-regulation of ODC or γ-GCS or TryR is responsible for increased T(SH)_2_ level in SAG resistant [58]–[60]. Additionally, other enzymes of thiol cascade, such as TXN & TXNPx were also up-regulated in SAG resistant strains [61], [62]. Similarly, Amp B resistance in *Leishmania* parasites was also reported to be associated with increased expression of enzymes involved in thiol metabolism alongwith absence of ergosterol in the membrane of resistant parasites, upregulation of Amp B efflux, and ROS scavenging machinery, having cumulative effect for Amp B resistance in *L. donovani* [39]. We have investigated whether LdTryS is upregulated in resistant isolates and found that the *TryS* mRNA is upregulated in Amp B resistant isolates when compared with sensitive strains. The expression of LdTryS protein in the same isolates were also found to be 2–3 fold increased in Amp B resistant isolates which supports previous studies on Amp B resistance mechanism in *Leishmania* parasites.

In trypanosomatids changes in gene expression is an important response to oxidative stress and is mainly achieved at post transcriptional level [63]. There is a broad response to oxidants in cells; low concentration of ROS may play important physiological role in cellular signalling and proliferation [64]–[66], while high concentration may cause oxidative stress. The H_2_O_2_ detoxification requires NADPH, as reducing equivalent, which is also needed for proper functioning of T(SH)_2_ cascades and supplied through pentose phosphate pathway. The TXN and TXNPx are vital for H_2_O_2_ reduction by T(SH)_2_ [67], [68] whereas, TryS is essential for synthesis of T(SH)_2_. On H_2_O_2_ treatment, parasites upregulate TryS in a dose dependent manner (Fig. 8A) probably to enhance the synthesis of T(SH)_2_ which would reduce the effects of ROS, RNS, and metabolize ROOH. It was found that LdTryS expression decreased at 200 µM H_2_O_2_ because higher concentration of ROS production (Fig. 7B) may affect normal adaptive response machinery and induce apoptosis. The TryS upregulation may also increase the expression of other proteins of thiol metabolism which aids parasite survival in hostile environment. The rate limiting enzymes present upstream of TryS, specially ODC and γ-GCS are also up-regulated to supply the precursors, GSH and Spd, for T(SH)_2_ biosynthesis. Although the concentration of oxidized T(SH)_2_ is lower than reduced form in normal conditions but under oxidative stress, its concentration increases more than 5 times because reaction is favoured in forward direction to metabolize toxic products and ROS.

In contrast to all other components of the T(SH)_2_ system, TryS does not have any close relatives within vertebrates. We have characterized *L. donovani* TryS which is similar to previously identified TryS from other kinetoplastids and is likely to play same functions in the cell. TryS can serve as a good drug target in *L. donovani* because it is the only enzyme responsible for T(SH)_2_ synthesis in this parasite. Earlier, many proteins of thiol metabolism had been explored for the drug target, such as, TXN, TXNPx, TryR, ODC, and TryS [21], [69]. The TXN and TXNPx may not be very suitable choice due to interaction between these proteins, and they are not easily inhibited specifically by any small molecule or they interact with more than one partner proteins suggesting non-specific interaction [69]. TryR was considered the best choice for drug target earlier and lots of reversible and irreversible inhibitors were tested *in vitro*. However, due to limited *in vivo* activity of reversible TryR inhibitors and the concern about possible side effects of the irreversible ones, none of the TryR inhibitors has so far entered into clinical trials [69]. Similarly, ODC inhibitors DFMO (Difluoromethylornithine) [37], [38], [70] was used both *in vitro* and *in vivo* but could not turn out as a drug against *Leishmania*, although DFMO (eflornithine) is an excellent drug against African trypanosomes. Therefore, most attractive drug target of the system appears to be TryS. It is a rather unique protein that apart from motifs reminiscent of ATP binding site, does not have any significant sequence similarity to any known mammalian proteins [24], [71]. So far, precise function and 3D structure has been studied only in *L. major* [24] and the reported structure was without any substrate, as well as, also lacks loop regions that are mechanistically very important. Recently, docking and molecular dynamic simulations studies has led to further insights into T(SH)_2_ biosynthesis and explains mode of substrate binding [72]. The TryS potential inhibitors obtained from a large scale library screening showed some active molecules specially phenyl substituted thiazoles, tetrazoles, and indazoles compounds that inhibited TryS of *T. brucei* in micromolar range and showed lower cytotoxicity [73]. The anti-parasitic activity of the lead compounds was shown by decreased intracellular concentration of T(SH)_2_ and Gsp, and an increased level of GSH, which mimics TryS knock-down of *T. brucei*. However, still only a few compounds have been tested against TryS in trypanosomatids and their chemical properties, remain poorly understood. Recently, some compounds (tomatine, conessine, uvaol, and betulin) were tested against purified LdTryS of non-pathogenic strain (UR6) and found to be competitive inhibitors with Spd. However, mode of inhibition with GSH and ATP was allosteric and all the inhibitors showed significant anti-leishmanial activity [22]. We achieved a far superior yield of purified rLdTryS compared to characterized TryS of *T. brucei*, *T. cruzi* and *L. major*. This may facilitate cost effective and high throughput screening of a larger number of compounds. In addition, *K*_m_ value of rLdTryS will be helpful in screening of inhibitors against LdTryS for rationale drug designing. Our present study demonstrated that the LdTryS plays a role in antioxidant mechanism and Amp B resistance.

## Supporting Information

Figure S1 **Drug sensitivity profile of sensitive strains and resistant isolates**. 1×10^6^ parasites were subjected to increasing concentration of drugs for 24 hours and cell viability determined by counting viable cells on a haemocytometer by trypan blue exclusion method. Sodium stibogluconate (SAG) concentrations ranging from 0 to 200 µg/ml were used for sensitive strains (A) and from 0 to 700 µg/ml for resistant isolates (B). Amphotericin B (Amp B) concentrations from 0–200 ng/ml were used for sensitive strain (C) and from 0–300 ng/ml for resistant isolates (D). A representative result of three independent experiments in duplicate is shown here.
(TIF)
