# Supplementary figures and images for: Stage-Dependent Expression and Up-Regulation of Trypanothione Synthetase in Amphotericin B Resistant Leishmania donovani
Source: PLoS One. 2014 Jun 5;9(6):e97600. doi: 10.1371/journal.pone.0097600 (PMC4046939; doi:10.1371/journal.pone.0097600)

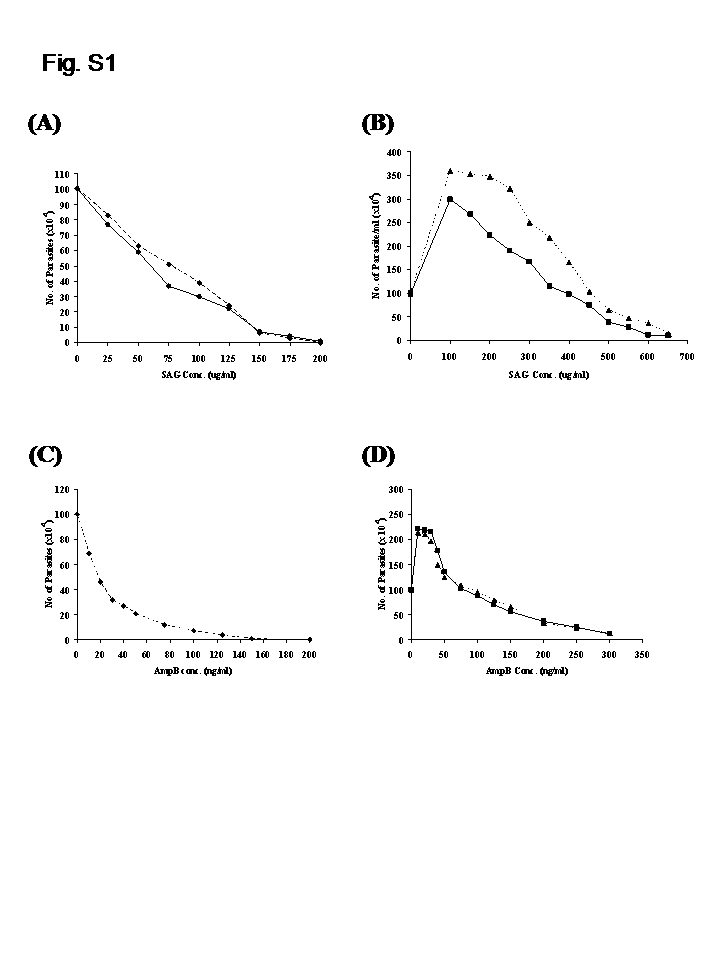

Supplement: Figure S1 — Drug sensitivity profile of sensitive strains and resistant isolates. 1×10^6^ parasites were subjected to increasing concentration of drugs for 24 hours and cell viability determined by counting viable cells on a haemocytometer by trypan blue exclusion method. Sodium stibogluconate (SAG) concentrations ranging from 0 to 200 µg/ml were used for sensitive strains (A) and from 0 to 700 µg/ml for resistant isolates (B). Amphotericin B (Amp B) concentrations from 0–200 ng/ml were used for sensitive strain (C) and from 0–300 ng/ml for resistant isolates (D). A representative result of three independent experiments in duplicate is shown here. (TIF) [file pone.0097600.s001.tif]
